# Supplementary material for: Deletion of wfs1 Impairs Oligodendrocyte Precursor Cells Dorsal Distribution and Myelination Through the wfs1-hmgcs1 Axis in Zebrafish
Source: Biology (Basel). 2026 Aug 16;15(16):1402. doi: 10.3390/biology15161402 (PMC13510151; doi:10.3390/biology15161402)
Supplement: Supplementary file 1 [file biology-15-01402-s001.zip › File S1-Transcriptome sequencing data and differentially expressed genes (DEGs) in wfs1-- zebrafish at 5 dpf/2.KEGG/wfs1KOvsWT/ALL/src/dre00830.html]

dre00830


- dre:116517

- Up regulated genes

116517(5.15873267159645)
- dre:393427

- Down regulated genes

393427(-1.25663853074503)

- dre:393580

- Up regulated genes

393580(1.34154931476468)

- dre:553239

- Down regulated genes

553239(-2.69220217535971)

- dre:562840

- Up regulated genes

562840(2.9733567031078)

- dre:407663

- Down regulated genes

407663(-1.06564682717154)

- dre:553239

- Down regulated genes

553239(-2.69220217535971)

- dre:553969

- Up regulated genes

553969(1.15424087647056)

- dre:562840

- Up regulated genes

562840(2.9733567031078)
- dre:407663

- Down regulated genes

407663(-1.06564682717154)

- dre:445083

- Up regulated genes

445083(5.52673269190901)

- dre:553969

- Up regulated genes

553969(1.15424087647056)

- dre:553969

- Up regulated genes

553969(1.15424087647056)

- dre:553969

- Up regulated genes

553969(1.15424087647056)

- dre:406731

- Down regulated genes

406731(-3.65881284610119)
- dre:678597

- Down regulated genes

100415796(-3.10645904576552)
- dre:570913

- Down regulated genes

570913\_1(-2.18577817117659)
- dre:767756

- Down regulated genes

767756(-1.81780175682628)

Close
